# Mouse and Rat Oxygen-Induced Retinopathy Models to Study Vascular Features Seen in Retinopathy of Prematurity

**DOI:** 10.21769/BioProtoc.5771

**Published:** 2026-08-05

**Authors:** Morgan Paige Tankersley, Shreya Beri, Aniket Ramshekar, Bright Asare-Bediako, Neal Sandeep Shah, James Regun Karmoker, Heng-Chiao Huang, M. Elizabeth Hartnett

**Affiliations:** 1 Byers Eye Institute, Department of Ophthalmology, Stanford University School of Medicine, Stanford, CA, USA; 2 Department of Ophthalmology, Chang Gung Memorial Hospital, Chiayi, Taiwan

**Keywords:** Oxygen-induced retinopathy, Vascular biology, Retinopathy of prematurity, Retinal dissection, Retinal flat mounting, Lectin immunostaining

## Abstract

Retinopathy of prematurity (ROP), a retinovascular disease, is a leading cause of childhood blindness worldwide. Given the constraints of studying molecular mechanisms in preterm infants, reproducible animal models are important to understand ROP pathophysiology. Mouse and rat oxygen-induced retinopathy (OIR) models are the most commonly used and recapitulate key vascular features seen in ROP. However, these models are susceptible to inherent variability that limits reproducibility, including inter-litter variability, consistency of oxygen delivery across experiments, retinal dissection technique, and immunohistochemistry. Here, we describe a comprehensive protocol for performing the most common mouse and rat OIR models, and procedures such as eye enucleation, retinal dissection and flat mounting, isolectin GS-IB4 staining, whole retina stitched fluorescence imaging from Z-stacks, and quantification of vascular features. This protocol provides important materials and procedural details to increase the reproducibility of the mouse and rat OIR models.

Key features

• Rat and mouse oxygen-induced retinopathy (OIR) models.

• Eye enucleation of experimental rat and mouse pups.

• Retinal flat mounting and immunostaining for rat and mouse eyes.

• Image analysis of retinal flat mounts from rat and mouse eyes.

## Graphical overview


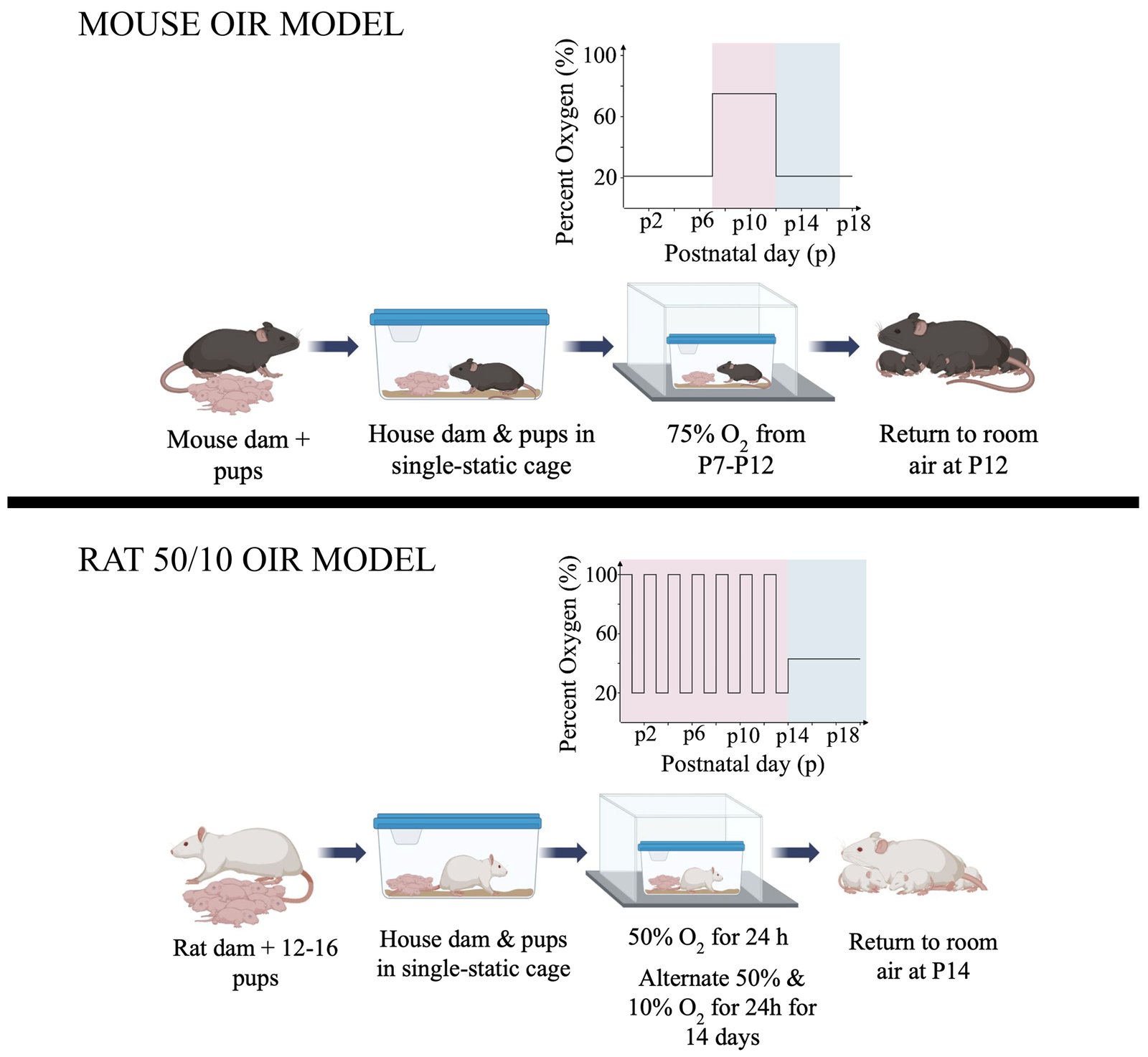


## Background

Retinopathy of prematurity (ROP), a retinovascular disease, is a leading cause of childhood blindness [1,2] and is described by a refined two-phase disease process [3,4]. Phase I involves impaired intraretinal vascular development, resulting in avascular retina and tissue hypoxia. Phase II involves pathologic intravitreal neovascularization (IVNV), which can lead to retinal detachment and permanent vision loss. Therefore, studies are warranted to understand ROP pathophysiology and identify potential therapeutic targets.

Studying molecular mechanisms of ROP in human preterm infants is not safe; as such, animal models are used to understand pathophysiology and investigate potential therapeutic targets. Oxygen-induced retinopathy (OIR) models have been used to study ROP pathophysiology [5–9], with the mouse and rat 50/10 OIR models being commonly used [9,10]. Mouse OIR recreates the vaso-obliteration of phase I and the vaso-proliferation of Phase II [3,10]. The rat 50/10 OIR model describes the refined hypothesis [4] of delayed physiological retinal vascular development, compromised vascularity (phase I), and vaso-proliferation as IVNV (phase II) [9]. However, model variability from inter-litter differences, consistency of oxygen delivery across experiments, incomplete removal of the hyaloid and vitreous affecting immunostaining, and loss of avascular peripheral retina during dissection in the rat model affect results and interpretation. While factors such as inter-litter variability are difficult to avoid and can only be controlled by including both experimental and control pups within each litter, other confounders can be minimized through reproducible experimental protocols. Here, we outline standardized protocols for (i) induction of mouse and rat OIR using a controlled OxyCycler system, (ii) retinal dissection and flat-mount preparation to ensure consistent staining, (iii) image acquisition capturing the entire retinal vasculature, and (iv) quantitative analysis of total retinal area, avascular area, and IVNV.

## Materials and reagents

**Biological materials**

1. Mice: Timed-pregnant C57BL/6J (Jackson Laboratories, strain number: 000664) or specified transgenic model(s)

2. Rats: Timed-pregnant Sprague Dawley dams (Charles Rivers, strain number: 001)

**Reagents**

1. Paraformaldehyde (PFA), 4% (Electron Microscopy Sciences, catalog number: 1573520S1L)

2. Phosphate-buffered saline (PBS), 10× (Thermo Fisher Scientific, catalog number: AAJ75889)

3. Triton X-100 stock solution 20% v/v (Sigma-Aldrich, catalog number: T9284)

4. Normal horse serum (Thermo Fisher Scientific/Invitrogen, catalog number: 31874)

5. Isolectin GS-IB4 from *Griffonia simplicifolia*, Alexa Fluor 488 conjugate (ThermoFisher Scientific, catalog number: I21411)

6. 75% ethanol (Gold Shield Distributors, catalog number: 412811)

7. Fluoromount-G mounting medium (Invitrogen, catalog number: 50-187-88)

8. Triamcinolone acetonide (400 mg per 10 mL) (Teva Pharmaceuticals, catalog number: NDC 0703-0245-01)

**Solutions**

1. 1× PBS (see Recipes)

2. Blocking buffer (see Recipes)

3. Lectin staining solution (see Recipes)

**Recipes**

**1. 1× PBS**

Dilute 10× PBS to 1× PBS with distilled water. Standard room temperature storage.

**2. Blocking buffer**

1× PBS

10% normal horse serum

0.5% Triton X-100

We recommend preparing fresh blocking buffer; however, we found that storing at 4 °C for up to 1 week will not affect tissue quality or staining. Warm blocking buffer to room temperature and mix before use.

**3. Lectin staining solution**

Dilute isolectin GS-IB4, Alexa Fluor 488 in blocking buffer, 1:100. Prepare fresh (recommended) and protect from light.

**Laboratory supplies**

1. 96-well plates (Genesee Scientific, catalog number: 25-109) (optional)

2. 1.5 mL microcentrifuge tubes (CELLTREAT, catalog number: 229442)

3. Kimwipes (Fisher Scientific, catalog number: 06-666)

4. Transfer pipettes (Fisher Scientific, catalog number: 13-711)

5. Superfrost Plus microscope slides (Fisher Scientific, catalog number: 1255015)

6. Coverslips/micro cover glass No. 1.5 (VWR, catalog number: 48393-151)

7. Coverslip weights (Mettler Toledo, catalog number: 01-912-181)

8. Gloves (Fisher Scientific, catalog number: 19149863)

9. Falcon^TM^ standard tissue culture dishes 21.29 cm^2^ (Corning, catalog number: 08-772B)

## Equipment

1. OxyCycler (Biospherix, model: A-84XOV) with OxyCycler software

2. Medical-grade oxygen tank, size K (Linde, catalog number: OX M-K) (or equivalent)

3. Medical-grade nitrogen tank, size K (Linde, catalog number: NI M-K) (or equivalent)

4. Portable Combustion Analyzer (Bacharach, model: 0024-8511-Fyrite InTech)

5. Stereomicroscope (Leica, model: EZ4 W)

6. Fluorescence microscope for tiled imaging and stitching (Keyence, model: BZ-X800)

7. Dumont #5 blunt straight tip forceps (FST, catalog number: 11251-30)

8. Fine grasping forceps (FST, catalog number: 11090-10)

9. Iris forceps: Bonn iris suture forceps (Titan Medical, catalog number: TMF612.50)

10. Curved tip forceps (Fisher Scientific, catalog number: 16-100-122)

11. Micro scissors: Noyes micro scissors (WPI, catalog number: 503306)

12. Platform Rocker (Corning, product number: 6702)

13. 30 G insulin needles (BD, SKU 305106)

## Software and datasets

1. Fiji (ImageJ) (version: 1.54p)

2. Keyence BZ-X800 imaging software (version: 01.03.00.01)

3. Keyence BZ-X800 Analyzer software (version: 1.1.2.4)

4. Microsoft Excel (version: Microsoft Office LTSC Professional Plus 2021)

## Procedure

*Note: For all described procedures, use 75% ethanol as needed to sanitize tools, workstations, and any other necessary surfaces.*

**A. Oxygen-induced retinopathy (OIR) models**

**A1. Mouse OIR (75% O_2_ from P7 to P12)**

1. Obtain timed-pregnant dams or set up breeding pairs to generate offspring of the desired genotype(s).

2. Assign P0 as the day of birth.

*Note: Remove the male breeder prior to delivery to reduce the risk of pup loss.*

3. House one dam with litter per cage (single-dam cage) with standard bedding, chow, and water ad libitum.

4. On P7 (7 days after birth, based on the recorded birth date/time), weigh pups and transfer the dam and litter into the OxyCycler chamber pre-equilibrated to 75% O_2_.

*Note: We do not exclude pups from the mouse OIR model based on weight or litter size; however, we record the number of pups and their weights to assess potential effects of experimental interventions [11].*

5. Maintain chamber oxygen at 75% ± 1% continuously from P7 through P12 and record oxygen logs.

6. Monitor dams and pups regularly for health and distress while minimizing chamber opening time.

*Note: The OxyCycler chamber is transparent, allowing routine visual monitoring without opening the chamber. Depending on cage type and placement, checks may be performed without interrupting oxygen control.*

7. In our experience, cages are not changed during P7–P12 hyperoxia. If a cage change or other intervention is required (e.g., dam/pup health concerns, experimental treatment, etc.), document the reason, timing, and duration of chamber opening.

8. On P12, remove the dam and litter from the chamber and return to room air (21% O_2_).

9. Record pup weights and the general health of the dam and pups.

*Note: Soda lime is not used for mouse OIR in this protocol.*

**A2. Rat 50/10 OIR (alternating 50% and 10% O_2_ from P0 to P14)**

1. Obtain timed-pregnant Sprague Dawley rat dams from Charles River.

**CRITICAL:** This model requires 12–16 pups per dam. To reliably meet this requirement, we recommend ordering additional timed-pregnant dams so pups can be supplemented, if needed (see **A9**).

2. Define P0 based on the observed birth time of the first pup as closely as possible and record the time.

3. Adjust each dam’s litter to 12–16 pups *before initiating oxygen cycling* (see **A9**). Tattoo supplemental pups for identification (see **A9a–b**).

4. House each dam with litter in a single-dam static cage with sufficient food and water.

5. Place the dam along with litter into the OxyCycler and initiate the rat 50/10 OIR model within 4–6 h of birth: 50% O_2_ for 24 h, alternating with 10% O_2_ for 24 h, for 14 days, followed by return to room air at P14. Maintain oxygen at ±0.1% throughout.

6. During oxygen cycling, perform daily monitoring.

**CRITICAL:** Count pups daily to ensure 12–16 pups remain in a litter. If pup mortality reduces litter size to under 12 pups, supplement litter (see **A9**). Briefly remove the cage from the chamber if needed for accurate counting.

**CRITICAL:** During the 10% O_2_ phase, verify accurate O_2_ level using an external calibrated sensor placed inside the running OxyCycler chamber and record verification results.

7. Change cages as required by institutional guidance and more frequently if condensation is observed. Minimize and document all chamber openings.

**Optional:** Mitigate CO_2_/humidity using soda lime as needed.

*Note: Perform chamber openings (including cage changes and pup counting) during the 50% O_2_ phase when possible, as this does not measurably alter vascular features.*

8. At P14, remove animals from the chamber (return to room air if not already) and record pup weights.

9. Litter size maintenance and exclusion criteria in rat 50/10 OIR:

Rationale: To promote competition for nursing and achieve postnatal growth restriction, which is an important contributor to the reproducible vascular phenotype in the rat 50/10 OIR model [12], 12–16 pups per dam are required throughout oxygen cycling.

a. Pup supplementation before starting 50/10 OIR model: If a dam delivers fewer than 12 pups, delay starting the 50/10 OIR model as long as the model can still be started within 4–6 h after the observed birth of the first pup. If a second litter is born within this window, transfer the pups from the newly born litter to bring the original litter size to 12–16 pups, and then place the supplemented litter into the OxyCycler chamber.

**CRITICAL:** Only pups placed into OIR within 4–6 h of birth are eligible for inclusion. Tattoo supplemental pups to allow identification during future analyses.

**CRITICAL:** If adequate supplementation cannot be achieved within the 4–6 h window, the litter may be excluded or used as room air supplemental pups.

b. Pup supplementation after starting 50/10 OIR model: If pup mortality reduces litter size below 12 pups after 50/10 OIR model has started, litter size may be supplemented to 12–16 pups using pups from room air litters to maintain nursing competition. Only add room air supplemental pups if close in age and size and clearly labeled as a late-added supplemental pup. Tattoo late-added supplemental pups for identification.

*Note: Avoid adding substantially larger pups, as they can over-compete and alter growth restriction and phenotype severity in the littermates. In our experience, room air pups are ~2 g heavier than OIR pups by P5; therefore, we do not use room air pups for supplementation after this age.*

c. If appropriate size matching is not possible, consider maintaining litter size using pups that were placed into OIR at the same time (e.g., transferring pups between concurrently run OIR litters initiated within the same birth window).

d. Analysis: Supplemental pups added before starting the 50/10 OIR model may be included in future analyses, but we recommend analyzing the data separately to ensure no confounders.

e. Grounds for litter exclusion:

i. Dams and litter are not placed into OIR within 4–6 h of the birth of the first pup.

ii. Supplemental pups added after starting the 50/10 OIR model (i.e., late-added supplemental pups) are excluded from analyses.

iii. If litter size drops below 12 pups and the laboratory cannot restore litter size with the above-mentioned approaches, consider excluding the litter from analysis and document the reason.

**B. Eye enucleation and fixation**

Typical collection timepoints: mouse P12 (avascular area, AVA) or P17 (AVA/IVNV); rat P14 (AVA) or P18–P20 (AVA/IVNV).

1. Euthanize animals in accordance with your lab’s and institutional IACUC protocols or local regulations.

2. Enucleate eyes using curved tip forceps, avoiding compression of the globe.

a. Gently proptose the eye by retracting the surrounding periorbital tissue.

b. Position the forceps gently beneath the sclera.

c. Slowly retract the forceps upward to free the globe, taking care not to puncture or compress it.

d. Sever the optic nerve to complete enucleation using micro scissors.

3. Puncture the cornea once with a 30G needle to facilitate fixative penetration.

4. Place one eye in a 1.5 mL microcentrifuge tube with 300–500 μL of 4% PFA.

5. Fix each eye in 4% PFA for 1 h at room temperature with gentle rocking.

6. Rinse eyes at least 3× in 1× PBS.

7. Eyes can be stored in 1× PBS at 4 °C until ready for dissection.

*Note: All steps and timepoints are identical for mouse and rat enucleation and fixation.*

**C. Retina dissection for mouse and rat flat mounts**

*Note: Perform dissections under a stereomicroscope. Keep tissue submerged in 1× PBS at all times. All steps are further visualized in Videos 1 and 2.*

**Figure 1.**
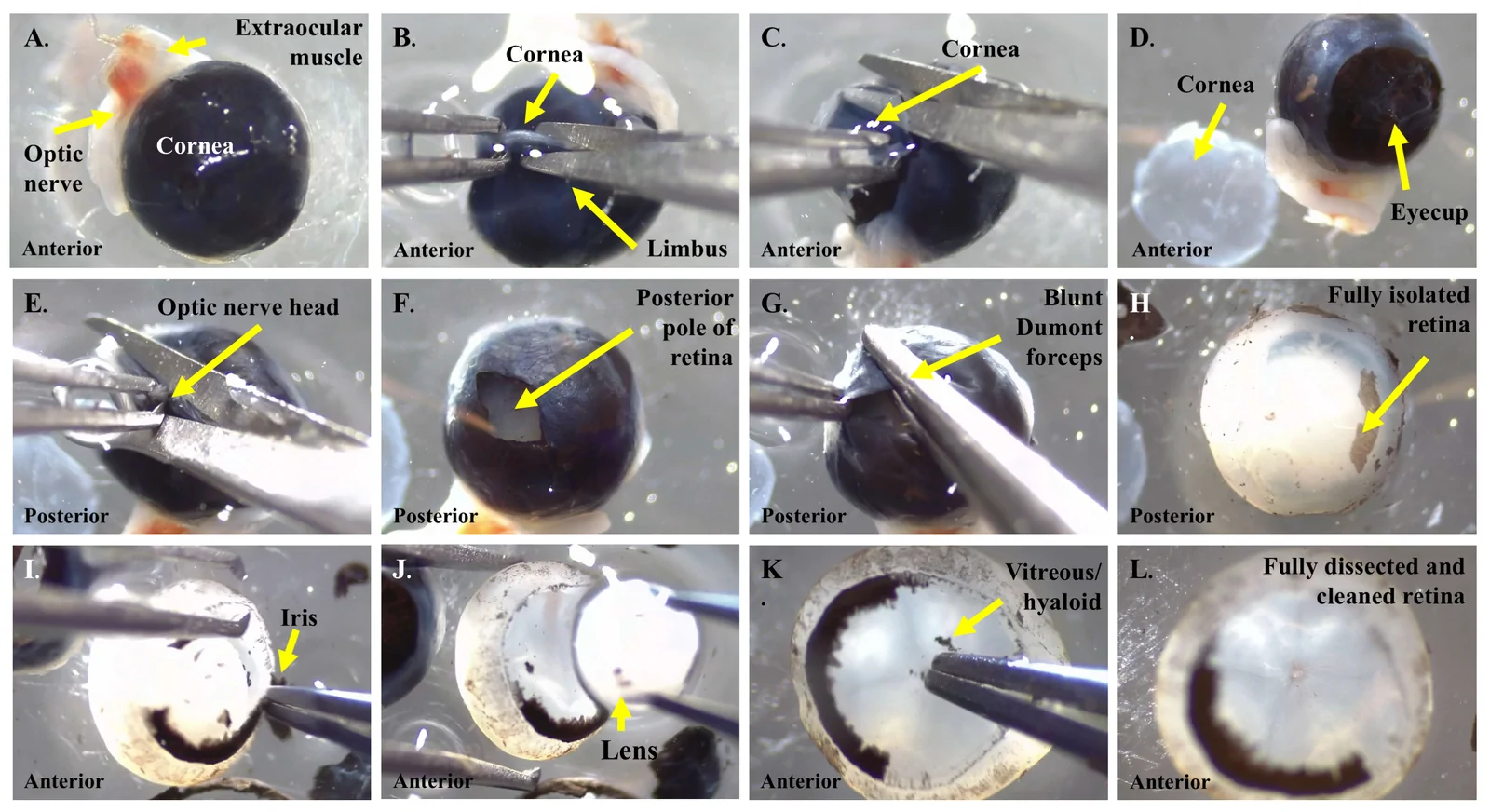
Mouse retinal dissection. (A) Fixed mouse eye in a Petri dish with 1× PBS. (B) Micro scissors making the initial limbal incision. (C–D) Circumferential incision above the limbus to separate the cornea from the eyecup. (E–F) Micro scissors making an incision at the optic nerve head. (G) Blunt Dumont forceps inserted into the incision plane. (H) Isolated retina with no tears/holes but expected residual retinal pigment epithelium (RPE) posteriorly. (I) Iris tissue removal from the ciliary body. (J) Iris suture forceps removing the lens. (K) Iris suture forceps removing the hyaloid and vitreous. (L) Final dissected and cleaned retina. All dissection steps are performed under a stereomicroscope (Leica EZ4 W).

1. Using a transfer pipette, place the fixed eye in a Petri dish containing 1× PBS (Figure 1A).

*Note: We have found that the transfer pipettes are of sufficient diameter to transfer the eye without harming the tissues. However, researchers may consider cutting the pipette tip if a larger diameter is needed.*

2. Using micro scissors, make a circumferential incision along the limbus and remove the cornea (Figure 1B–D).

3. Separate the retinal pigment epithelium (RPE)/choroid/sclera complex from the neural retina:

a. Place the eye with the cornea facing down. Using grasping forceps, gently hold the optic nerve head. With micro scissors, cut away the optic nerve head along with the attached choroid/sclera at the base of the eye (Figure 1E–F).

b. Insert the closed tips of blunt Dumont forceps into the incision plane just under the RPE/choroid/sclera without tearing the posterior retina (Figure 1G).

c. Advance laterally or circumferentially using sweeping motions to separate the neural retina from the underlying RPE/choroid/sclera.

d. Continue until the RPE/choroid/sclera complex is fully detached and removed (Figure 1H).

4. Inspect the isolated retina and trim away any remaining anterior segment tissue (e.g., residual iris) using grasping forceps (Figure 1I).

5. Remove the lens carefully using iris suture forceps without pulling on the retina (Figure 1J).

6. Remove hyaloid/vitreous remnants (Figure 1K):

a. Using iris suture forceps, grasp the hyaloid membrane at an attachment point.

b. Peel it away from the inner retinal surface using slow, controlled traction.

c. Avoid contacting or scraping the retina with forceps tips.

7. Store the isolated retinal eyecup in 1× PBS at 4 °C until flat mounting.

**Figure 2.**
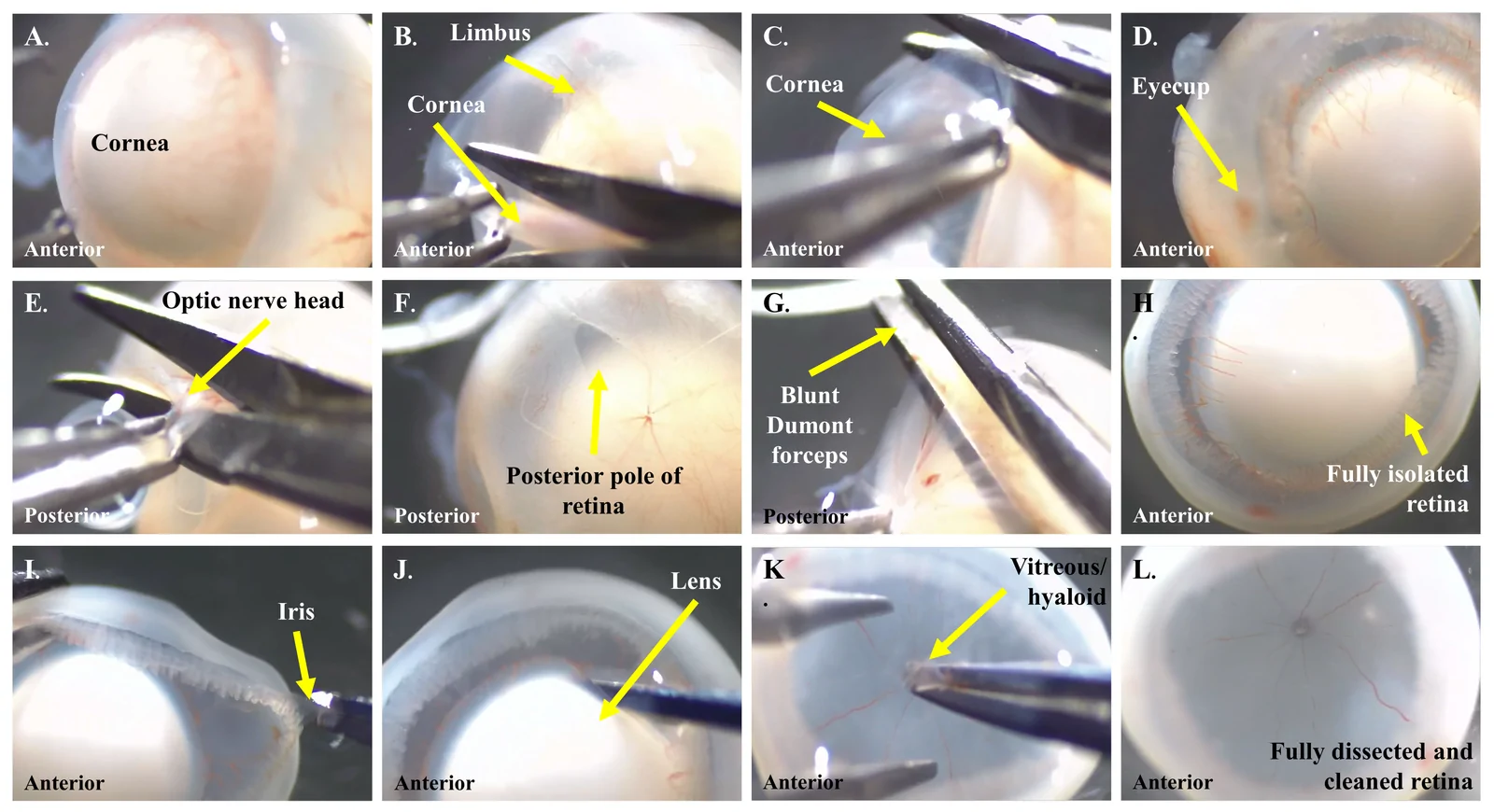
Rat retinal dissection. The albino rat eye lacks pigmentation; however, the dissection procedure is similar to that of mice (see Figure 1). (A) Eye in PBS. (B) Circumferential incision. (C) Cornea removal. (D) Eyecup. (E) Posterior incision. (F) Completed incision. (G) Retina separation. (H) Isolated retina. (I) Trim anterior tissue. (J) Lens removal. (K) Vitreous removal. (L) Final retina.

*Note: The dissection procedure is identical for mouse and rat. Panels in Figure 1 (mouse) correspond directly to panels in Figure 2 (rat) (i.e., Figure 1A = Figure 2A, etc.). The rat eye is larger and easier to visualize. However, the albino rat eye lacks pigmentation, making the sclera and RPE/choroid layers difficult to distinguish from the retina visually during dissection.*

**Video 1.**
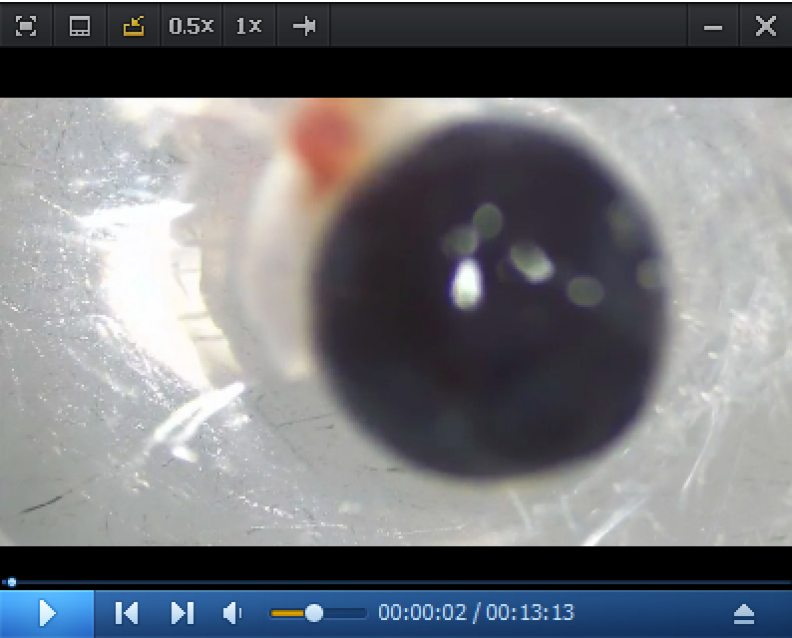
Mouse retina dissection

**Video 2.**
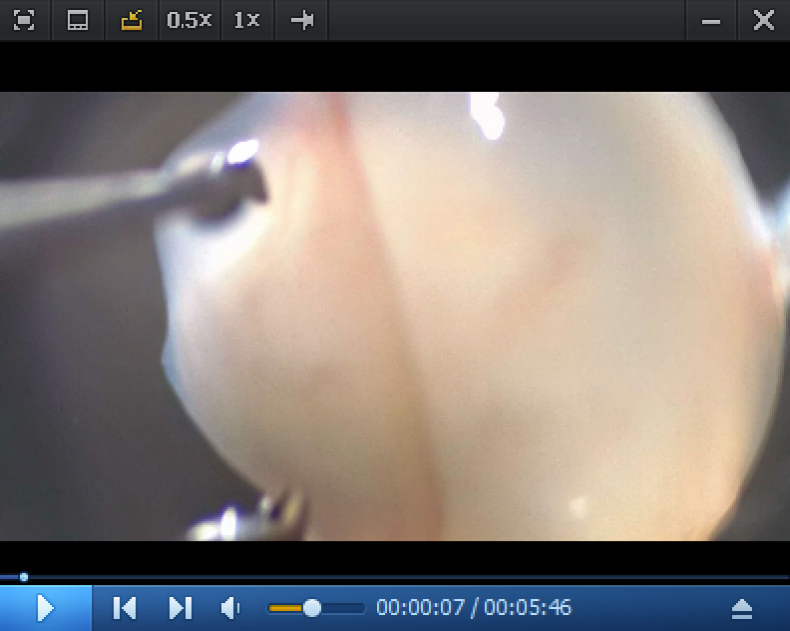
Rat retina dissection

**C1. Triamcinolone-assisted vitreous visualization (Optional)**

**Figure 3.**
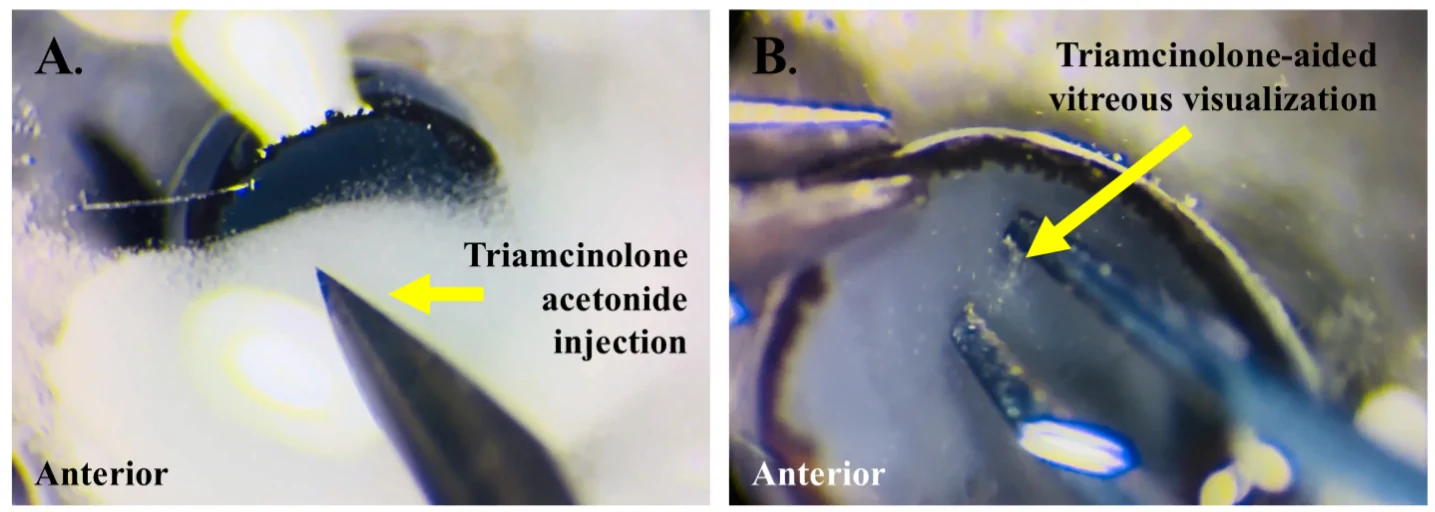
Enhanced vitreous removal via triamcinolone-assisted visualization. (A) Representative image depicting triamcinolone acetonide placement into the posterior eyecup. (B) Triamcinolone-stained vitreous removed by iris suture forceps.

Incomplete vitreous removal reduces antibody penetration and increases imaging artifacts due to trapped debris and precipitates that appear as nonspecific staining. Triamcinolone acetonide may be used to help visualize the transparent vitreous and facilitate complete removal (Figure 3). In our experience, triamcinolone was used to help train new laboratory members in removing the vitreous.

*Note: Triamcinolone is added, and the vitreous/hyaloid is removed, before separating the neural retina from the sclera/RPE/choroid complex, as the dark pigmentation of this tissue provides contrast for visualizing the white triamcinolone crystals. However, if using albino rats, the unpigmented sclera/RPE/choroid complex is transparent, making visualization of the triamcinolone-coated vitreous difficult.*

1. Apply ~50–100 μL to the retinal surface for ~30 s.

2. Rinse at least twice with 1× PBS before proceeding.

**D. Isolectin staining of retinal flat mounts**

1. Staining preparation: Place each retina in an individual well (e.g., 96-well plate) containing 100–300 μL of 1× PBS.

2. Blocking/permeabilization: Remove 1× PBS and incubate retinas in 100 μL of blocking buffer for ≥1 h at room temperature with gentle rocking.

*Note: Prepare fresh buffer daily to improve staining quality.*

3. Isolectin incubation:

a. Prepare lectin staining solution.

b. Remove blocking buffer and incubate retinas in 100 μL of lectin staining solution overnight (12–18 h) at 4 °C with gentle rocking, protected from light.

4. The next day, wash retinas in 100–300 μL of 1× PBS at least three times for ≥10 min each at room temperature with gentle rocking, protected from light.

**E. Flat mounting of retinas**

1. Using a transfer pipette, transfer the stained retina, vitreous side facing upward, onto a Superfrost Plus slide with a small volume of 1× PBS.

2. Make four equidistant radial relief cuts from the periphery toward (but not through) the optic nerve head to form four *petals*.

3. Use a Kimwipe to gently wick away excess PBS from around (not on top of) the tissue.

4. Gently flatten each flap against the slide so the retina lies as a single layer with minimal folds. Orient the retina with the RPE side against the slide and the retinal ganglion cell (RGC) layer facing up.

*Note: If needed, use a Kimwipe to wick fluid at the edge of the tissue to help the petal lay flat.*

5. Add mounting medium:

a. Apply 35–60 μL of mounting medium on a coverslip (22 mm × 22 mm per retina).

b. Gently lower the coverslip so that the drop of mounting medium contacts the flat-mounted retina and spreads evenly, displacing any air bubbles.

6. Place a ~10-g weight centered on the coverslip to flatten the retina and displace bubbles away from the retina.

7. Store slides protected from light with the weight in place until the mounting media is dry.

*Notes:*

*1. We usually wait ~30 min until the mounting media has sufficiently dried for the weight to be removed.*

*2. Peripheral bubbles may be acceptable; bubbles overlying vascular regions used for quantification might warrant exclusion. In our experience, using a larger volume of mounting medium (~60 μL) and ensuring it directly contacts the tissue during coverslip placement minimizes air bubble formation on the retina. Eyes/retinas with dissection errors that prevent accurate quantification (e.g., major tears, missing large retinal regions, severe folds, or artifacts obscuring AVA/IVNV) may be excluded.*

**F. Imaging (whole-retina stitched images)**

*Note: Image each retina under identical settings when possible.*

1. Use a Keyence BZ-X800 fluorescence microscope (or equivalent) to acquire a whole-retina tile scan.

2. Objective: 11× (or similar magnification as configured by the microscope system being used).

3. Exposure (typical for isolectin AF488): 1/25 s (adjust only if required to avoid pixel-level saturation).

4. Z-stacking: define upper and lower imaging limits for depth by scrolling through the different vascular layers.

5. Stitching: 7 × 7 grid using Keyence stitching function.

6. Save the stitched image as a TIFF (preferably 16-bit) with a consistent naming convention, for example: **Species_Model_Timepoint_LitterID_AnimalID_Eye(R/L)_Date.tif.**

7. Use BZ-X800 OME-TIFF Converter software to generate standardized file types with preserved metadata, enabling downstream stitching and analysis in Fiji (ImageJ).

8. Individual tiles are automatically detected, organized into grids, and stitched using the Grid/Collection StitchingFiji (ImageJ) plugin with linear blending and subpixel alignment.

9. Stitched images are split into individual channels, assigned pseudo-colors, and subjected to maximum intensity projection.

10. Final composite images are generated by merging channels and saved for quantification.

11. Quality control:

a. Exclude images with major tears through the optic nerve head region, large folds obscuring substantial retina, widespread staining failure, or bubbles obscuring quantification regions.

b. Record exclusions and document reason.

## Data analysis

**A. Quantification of total retina (TR), avascular area (AVA), and IVNV in Fiji (ImageJ)**

**A1. File setup**

1. Open the stitched TIFF image in Fiji (*File* → *Open*).

2. Confirm spatial scale metadata is present; if not, set scale using microscope calibration.

3. If needed for visualization only, adjust brightness/contrast (Ctrl+Shift+C on Windows or Command+Shift+C on Mac) as needed.

*Note: Avoid over exposing pixels in vascular regions used for quantification; if exposure differs from previous experiments, record exposure time in the filename or metadata log.*

**A2. Total retina (TR) region of interest (ROI)**

**Figure 4.**
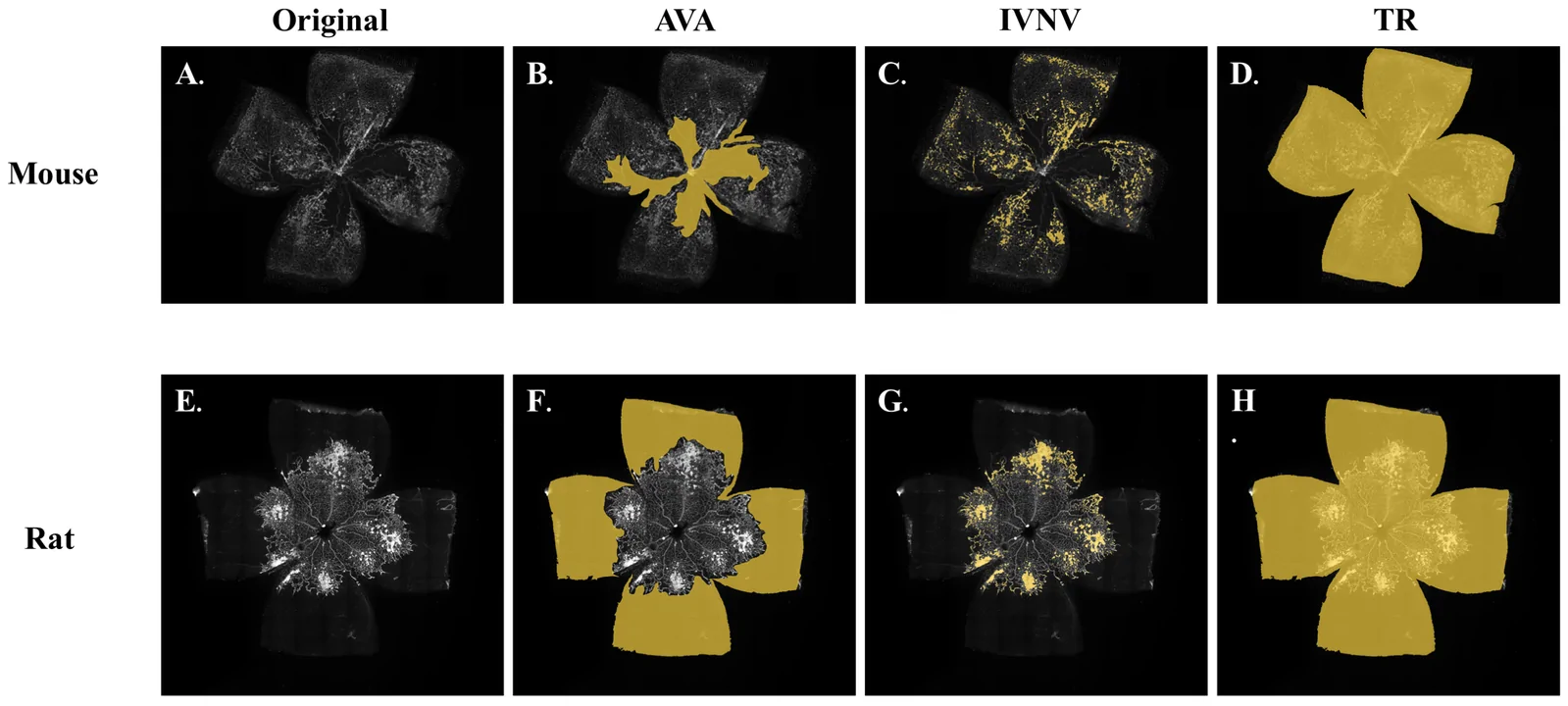
Representative retinal flat mount images from mouse and rat oxygen-induced retinopathy (OIR) models marked for vascular features of interest. Yellow color overlays indicate regions of interest (ROIs) used for quantitative analysis. **(A–D)** Mouse OIR. **(A)** Lectin-stained retinal flat-mount image (original). **(B)** Avascular area (AVA) marked within the central region of the retina. **(C)** Intravitreal neovascularization (IVNV) highlighted across the retina. **(D)** Total retinal area (TR) encompassing the entire flat mount. **(E–H)** Rat OIR. **(E)** Original flat-mount image. **(F)** AVA marked within the peripheral region of the retina. **(G)** Intravitreal neovascularization (IVNV) highlighted across the retina. **(H)** Total retinal area (TR) encompassing the entire flat mount.

1. Using the *Wand* (tracing) tool or *Freehand/Polygon* selection:

a. Outline the entire retinal boundary (include all four petals) (Figure 4B, H).

2. Add the selection using keyboard shortcuts (t or Ctrl+t) to the Region of Interest (ROI) Manager in ImageJ and rename as total retina (TR).

**A3. Avascular area (AVA) ROI**

1. Identify the avascular retina based on the absence of lectin-positive vessels.

a. Mouse: AVA is typically central (Figure 4B).

b. Rat: AVA/delayed vascularization is often peripheral (Figure 4F).

2. Trace the avascular region(s) using the *Freehand* or *Polygon* selection tool.

3. Add to ROI Manager using keyboard shortcuts (t or Ctrl + t) in ImageJ and rename as AVA.

**A4. IVNV ROIs**

1. Reset brightness/contrast, then reduce the maximum until IVNV tufts are clearly distinguishable from in-plane vessels.

2. Identify IVNV based on morphology (Figure 4C, G).

*Note: Exclude large in-plane vessels and obvious artifacts (e.g., fold edges, debris, hyaloid remnants).*

3. Select IVNV by manually outlining each tuft using the *Freehand selection* tool and add each ROI to the ROI Manager (e.g., IVNV_01, IVNV_02, …) using keyboard shortcuts (t or Ctrl+t).

4. Combine IVNV ROIs (optional): In ROI Manager, select all IVNV_*ROIs and use *More* → *Combine* to create a single combined IVNV_totalROI (or keep separate and sum areas in Excel).

**A5. Measure and export**

1. Configure measurements in Fiji: Navigate to *Analyze* → *Set Measurements* and select *Area*.

2. Measure ROIs: Measure the total retina (TR), avascular area (AVA), and each IVNV ROI (IVNV_*), or the combined IVNV_total ROI if used.

3. Save ROIs: In ROI Manager, select *More* → *Save…* and save all ROIs as a .zip file using the same name as the image.

4. Export measurements (*Results* window) as .csv.

**B. Data processing (Excel) and reported outcomes**

1. Import ROI area measurements into Excel.

2. For each retina, tabulate:

a. TR_area

b. AVA_area

c. IVNV_area (sum of all IVNV_* if measured individually)

3. Calculate:

a. %AVA = (AVA_area/TR_area) × 100

b. %IVNV = (IVNV_area/TR_area) × 100

4. Recommended minimal reporting table columns:

a. Species, Model, Timepoint, LitterID, AnimalID, Eye (R/L), Biological Sex (if tracked), TR_area, AVA_area, IVNV_area, %AVA, %IVNV, Notes/QC flags.

5. For statistical analysis, we recommend using a mixed effects linear regression model to account for pups nested within the same litter. If both eyes are analyzed, include random effects for eyes nested within the same animal and animals nested within the same litter.

## Validation of protocol

Original research papers in which the mouse and rat OIR models were described:

- Smith et al. [10]. Oxygen-induced retinopathy in the mouse. *Investigative Ophthalmology & Visual Science*.
- Penn et al. [13]. Oxygen-induced retinopathy in the rat: relationship of retinal nonperfusion to subsequent neovascularization. *Investigative Ophthalmology & Visual Science*.

## General notes and troubleshooting

The mouse OIR and rat 50/10 OIR models are widely used to investigate the pathophysiology of ROP. Their respective strengths and limitations are summarized in Table 1, while common challenges encountered during the protocol and corresponding troubleshooting strategies are provided in Table 2.

**Table 1.** Strengths and weaknesses of the mouse and rat 50/10 OIR models. Data from both the mouse and rat OIR models provide strong mechanistic insight into ROP.

Model	Strengths	Weaknesses
Mouse OIR model	Commonly used and standardized [10]. Models IVNV (phase II) and may translate features seen in aggressive ROP [10]. Used to study molecular mechanisms using transgenic mice (e.g., tamoxifen-inducible Cre-loxP knockout models).	The inner plexus is already vascularized to the peripheral retina at P7, restricting the study of delayed intraretinal vascularization to the peripheral retina.
Rat 50/10 OIR model	Commonly used and standardized [9]. Pups are exposed to fluctuating oxygen levels, which mimic oxygen variability observed in preterm infants with severe ROP [13]. Models delayed intraretinal vascularization toward the peripheral retina, resulting in both peripheral avascular retina (phase I) and IVNV (phase II) [9] Rat pups demonstrate reduced weight gain due to litter size restriction (12–16 pups per litter) [12], a known risk factor for severe ROP in humans [14]	Time-intensive and sensitive. Rat pups must be placed into the OIR model within 4–6 h after birth. Difficult to study molecular mechanisms, but can be addressed using cell type–specific knockdown of signaling effectors by lentivirus-mediated shRNA expression in a microRNA context [15–17]. Intravitreal pharmacologic agents are used, but affects signaling in multiple retinal cell types

**Table 2.** Common problems and solutions. Troubleshooting guide for addressing issues encountered during tissue fixation, permeabilization, dissection, mounting, and imaging of retinal vasculature with lectin staining.

Problem	Possible causes	Solutions
Weak lectin signal	Under-fixation, incomplete permeabilization, old lectin	Confirm 4% PFA fixation time; ensure 0.5% Triton in block; prepare fresh buffers and lectin dilution; extend incubation to 18 h
High background	Insufficient washes, tissue damage, possible over-fixation	Increase PBS wash number/duration; ensure gentle handling; add an additional blocking step; optimize fixation duration
Retina tears during dissection	Tissue dried, excessive traction	Keep submerged; use minimal force; slow separation of layers near optic nerve head
Folds after mounting	Incomplete relief cuts, too much fluid under tissue	Ensure four radial cuts; wick PBS thoroughly before mounting; let retina partially adhere before coverslip
Bubbles over tissue	Coverslip lowered too fast, insufficient mounting medium	Lower coverslip at an angle; add mounting medium at the edge and allow capillary flow; re-mount if bubbles overlay key regions
IVNV difficult to distinguish	Contrast settings inconsistent, hyaloid remnants	Reset brightness/contrast setting before IVNV scoring; improve vitreous removal; use triamcinolone visualization
